# Driving Apart and Segregating Genomes in Archaea

**DOI:** 10.1016/j.tim.2016.07.001

**Published:** 2016-12

**Authors:** Daniela Barillà

**Affiliations:** 1 Department of Biology, University of York, York YO10 5DD, UK

**Keywords:** genome segregation, chromosome, plasmid, Archaea.

## Abstract

Genome segregation is a fundamental biological process in organisms from all domains of life. How this stage of the cell cycle unfolds in Eukarya has been clearly defined and considerable progress has been made to unravel chromosome partition in Bacteria. The picture is still elusive in Archaea. The lineages of this domain exhibit different cell-cycle lifestyles and wide-ranging chromosome copy numbers, fluctuating from 1 up to 55. This plurality of patterns suggests that a variety of mechanisms might underpin disentangling and delivery of DNA molecules to daughter cells. Here I describe recent developments in archaeal genome maintenance, including investigations of novel genome segregation machines that point to unforeseen bacterial and eukaryotic connections.

## Trends

Considerable diversity is observed in the features of the cell cycle of different members of the Archaea domain.

For the species characterized to date, a marked divide has emerged between monoploid Crenarchaea and polyploid Euryarchaea with regard to chromosome copy number.

SMC proteins appear to play an important role in chromosome segregation, although further studies are needed to substantiate their action and impact on the process.

Orthologs of bacterial ParA DNA partition proteins are widespread across Archaea and have been shown to be involved in chromosome segregation in synergy with archaea-specific factors.

Recent investigations have disclosed a three-component genome segregation machinery borrowing building blocks from Bacteria and Eukarya.

## Archaea: The Third Domain of Life

Archaea are the third branch of the tree of life [1]. Since their discovery 40 years ago, members of this domain have been isolated from a vast array of diverse ecological niches, including soil, ocean plankton, freshwater lakes, acidic hot springs, volcanic mud, deep-sea hydrothermal vents, and salty lakes. The first archaea to be analyzed were from extreme ecosystems, but they are now known to be ubiquitous on our planet. For example, it has been estimated that the world ocean contains approximately 1.3 x 10^28^ archaeal cells [2]. Their ubiquity and abundance suggest that archaea are key players in regulating global biogeochemical cycles on Earth. Archaea have generated also considerable interest because of their ability to adapt to life under extreme conditions, including high and low temperatures, very acidic and alkaline pH, and high salinity.

Sequences available for an increasing number of genomes (521 to date) together with genetic and biochemical studies have shown that Archaea exhibit a mosaic of features from the other two domains of life, Bacteria (e.g., energy generation, metabolism, transport, nitrogen fixation and CRISPR-*cas* systems) and Eukarya (e.g., DNA replication, transcription, translation, and protein folding). However, archaea are also characterized by unique molecular features such as methanogenesis and ether-linked isoprenoid lipid chains in their cell membranes [3]. Archaea are also interesting for studies on the origin of life: these microbes can be considered a ‘time capsule’ that provides a glimpse of what life may have been like on Earth when this was a planet bursting with geological activity billions of years ago. Members of the Archaea domain that have been studied to date fall into three main phyla: Crenarchaea, Euryarchaea and Thaumarchaea.

Despite the progress made in decoding molecular mechanisms in these organisms, very little is known about how the process of DNA segregation is organized in archaea and the subject remains a black box in this domain of life. This review focuses on recent developments in the area of archaeal genome segregation, discussing molecular machineries that have been recently identified and emerging trends.

## Genome Organization: Chromosomes, Megaplasmids, and Smaller Replicons

Like bacteria, archaea are prokaryotic cells whose genetic material is not confined by a membrane into a separate compartment. Archaeal genomes consist of a circular chromosome and often also large or small extrachromosomal elements. Virtually all the halophilic Euryarchaea sequenced to date harbour a 2.0–3.9 Mbp chromosome and multiple large plasmids [4]. For example, *Haloarcula marismortui* contains a 3.13 Mbp chromosome together with eight additional replicons of which the largest, pNG700, is 410 kbp. Many species are characterized by a dynamic flux between chromosome and plasmids which is facilitated by the presence of numerous insertion sequences that lead to episodes of integration of plasmids into the chromosome [5]. Accessory replicons also are found in Crenarchaea in the form of cryptic and conjugative plasmids, such as pNOB8 of *Sulfolobus* NOB8H2. However, crenarchaeal plasmids tend to be relatively small, with sizes below 50 kbp [6]. Unlike Bacteria that contain chromosomes with a single replication origin, and instead similarly to Eukarya, Archaea harbour a chromosome containing one or more replication origins 5, 7, 8, 9.

## Cell Cycle in Archaea: Different Strokes for Different Folks

Every cell goes through defined functional stages in the course of its lifespan, during which vital processes, such as growth of cellular structures, chromosome replication followed by segregation and division, take place in an ordered timeline. The start and end points mark the birth of new daughter cells, and the length of intervening time defines the generation or doubling time. The cell cycle in bacteria comprises three stages: the growth or B phase during which the cell actively synthesises proteins, lipids, and other building blocks in preparation for DNA duplication; the chromosome replication or C phase; and the postreplication or D period that terminates with cell division. A different terminology is adopted for the eukaryotic cell cycle which consists of the G1 (gap 1), S (DNA synthesis), G2 (gap 2) and M (mitosis) stages. This latter nomenclature has been most commonly used to describe the cell cycle of members of archaeal phyla. The knowledge built up so far indicates that archaea belonging to different lineages exhibit great variability in their cell cycle.

### Crenarchaea

Pioneering work by the Bernander group in the 1990s initiated a survey of the cell cycle in Crenarchaea. These investigations revealed that members of the thermophilic genus *Sulfolobus*, such as *Sulfolobus solfataricus* (doubling time ∼425 minutes) and *Sulfolobus acidocaldarius* (doubling time ∼213 minutes), are characterized by a brief G1 prereplication period that accounts for no more than 5% of the entire cell cycle [10]. The G1 ends with the inception of chromosome replication, S stage, which proceeds for 30–35% of the cell cycle and is followed by a very protracted G2 phase. This postreplicative interval occupies more than 50% of the cycle and is a defining hallmark of the crenarchaeal species investigated so far 10, 11. Members of the genus *Sulfolobus* are monoploid: the cells harbour one single chromosome in G1 stage and, upon replication, two copies are present. As the G2 stage is very prolonged, *Sulfolobus* species contain two chromosome copies for most of the cell cycle. During the G2 phase chromosomes become organized for segregation. Interestingly, both cytological and biochemical studies have indicated that the two chromosomes remain paired and connected for a prolonged time during G2 phase and appear as a single nucleoid in most cells 12, 13. Afterwards, during the M phase, chromosome segregation takes place very swiftly, followed in rapid succession by cell division. Chromosome segregation and cytokinesis occur in a time span equivalent to ∼10% of the cell cycle and appear closely interlinked.

**Figure 1:**
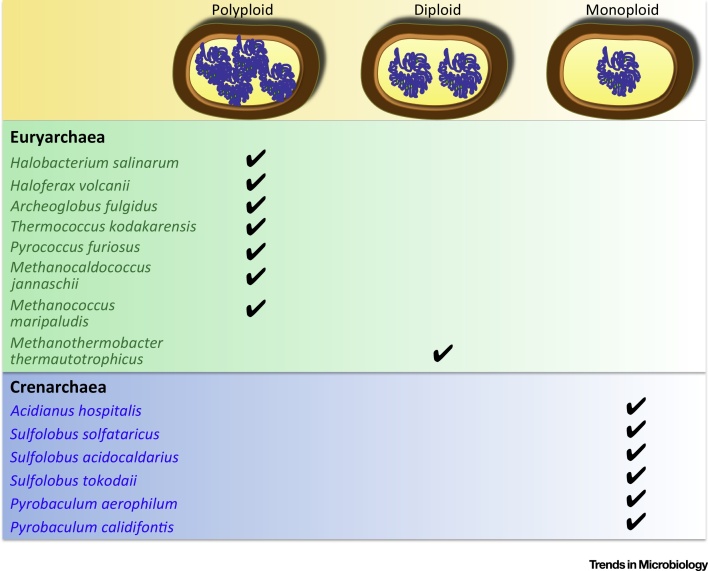
Ploidy in a Set of Characterized Members of the Euryarchaea and Crenarchaea Phyla. All the euryarchaeal species (green box) contain multiple chromosome copies, whereas the crenarchaeal species (blue box) harbour a single chromosome.

Analogous cell-cycle patterns and timing have been observed for all the Crenarchaea spp. studied to date which include *Sulfolobus tokodaii*, *Acidianus hospitalis*, *Aeropyrum pernix*, *Pyrobaculum aerophilum*, and *Pyrobaculum calidifontis* [11]. Although the number of crenarchaeal species characterized so far is limited, interestingly all are monoploid and harbour two chromosome copies only on completion of DNA replication (Figure 1). This observation suggests that an accurate and rigorous genome segregation mechanism must operate in these species to ensure the faithful distribution of chromosomes to daughter cells. Whereas chromosome segregation in bacteria occurs concomitantly with replication, the picture in Crenarchaea is very different: the two processes are temporally separated and genome segregation takes place only at the end of the protracted G2 stage 12, 13. The two *Pyrobaculum* spp. that have been examined represent a slight deviation from the canonical paradigm as chromosome segregation appears to be largely synchronized with replication [11].

### Euryarchaea

The Euryarchaea phylum includes a wide range of families that populate the most diverse niches and exhibit disparate lifestyles. The sulphate-reducing *Archeoglobus fulgidus* displays cell-cycle features resembling those observed for crenarchaeal *Sulfolobus* spp. [14].

In contrast, the methanogen *Methanocaldococcus jannaschii* is polyploid and characterized by a very relaxed cell cycle [15]. Cells in exponential phase accommodate between 3 and 15 chromosome copies that are reduced to a number between 1 and 5 in stationary phase. Cell division occurs asymmetrically, resulting in an uneven distribution of chromosome copies to daughter cells [15]. The apparent randomness and lack of order dominating these processes beg the question of whether a genome segregation system operates in *M. jannaschii* and whether the presence of multiple chromosome copies makes a DNA-partitioning apparatus dispensable. Another methanogen belonging to a different order, *Methanothermobacter thermautotrophicus*, grows as chains of rod-shaped cells each of which contains two chromosomes in the G1 phase which segregate soon after replication [16]. In this case the G2 stage is very brief or completely absent. *Methanococcus maripaludis* is an extreme example of polyploid euryarchaeon with as many as 55 chromosome copies [17].

Halophilic archaea are characterized by the presence of multiple copies of the chromosome (Figure 1). To some extent this feature has hindered a detailed dissection of their cell cycle. *Halobacterium salinarum* contains approximately 30 chromosome copies during exponential phase which are reduced to around 10 in stationary phase [18]. Interestingly, this archaeon does not have a temporally demarcated S stage, as DNA replication occurs throughout the cell cycle [19]. However, lack of a tight replication control does not result in a deregulated cell cycle, as shown by the complete block of cell division upon inhibition of DNA polymerase [20]. Furthermore, genome segregation mechanisms that deliver equal number of chromosomes to the two daughter cells appear to be in place in *H. salinarum* [18]. A different halophile, *Haloferax volcanii*, is also highly polyploid, with cells harbouring approximately 20 chromosome copies in exponential phase and approximately 12 during stationary phase [18]. The chromosome copy number of the euryarchaeon *Thermococcus kodakarensis* has also been recently investigated. Analogously to the situation in other members of this phylum, *T. kodakarensis* cells show polyploidy with a chromosome copy number fluctuating between 19 and 7 from exponential to stationary phase [21].

As summarized above, all Euryarchaea investigated to date are polyploid, with the exception of *M. thermautotrophicus* that is diploid. This is in sharp contrast with observations related to characterized Crenarchaea which are all monoploid (Figure 1) [17]. Accommodating and managing multiple chromosome copies raises a number of interesting biological questions, including the mechanism of DNA packaging within relatively small cells. A recently proposed hypothesis suggests a direct correlation between the presence of histones and polyploidy [21]. In fact, histones are commonly found in Euryarchaea, but not in Crenarchaea 22, 23.

### Thaumarchaea

The Thaumarchaea lineage was recognized as an independent phylum in 2008 and its members are widespread in terrestrial and ocean niches [24]. The cell cycle of the ammonia-oxidizer *Nitrosopumilus maritimus* shows a prereplication G1 phase that is longer than that observed for Crenarchaea and is equivalent to 19–29% of the full cell cycle. A very protracted S stage follows, which corresponds to 45–53% of the cycle: remarkably, 15–18 hours are necessary to replicate the chromosome. Genome segregation occurs quickly after replication termination, with the G2 phase being very short or absent [25].

Altogether, the studies on the archaeal cell cycle conducted so far have highlighted disparities and analogies among phyla and, interestingly, a dichotomy between monoploid and polyploid archaea has emerged (Figure 1). Cells harbouring a single chromosome copy need to employ a segregation mechanism to ensure an accurate distribution of the genetic material inherited by the progeny. The presence of multiple copies of the chromosome, sometimes as many as 55 as in *Methanococcus maripaludis* [21], raises the question as to whether polyploid archaea require a chromosome-sorting partition machine.

## Delivering Genomes to Daughter Cells: Snapshots from Bacteria

The molecular events and factors underpinning chromosome segregation in eukaryotes have been extensively investigated. During mitosis, the microtubules of the mitotic spindle capture sister chromatids and pull them to opposite spindle poles [26]. The mechanisms and proteins that drive chromosome segregation in bacteria are not fully elucidated; however, significant progress has been made in the past two decades. Interestingly, only a few complexes have been identified as key players in the process of bacterial DNA segregation.

### Moving DNA Molecules Apart: The ParABS System

Pioneering work by the Austin and Hiraga groups identified the ParAB module as responsible for the active partition of low-copy-number plasmids in *Escherichia coli* 27, 28. This system was later found to be encoded by most bacterial chromosomes [29] and consists of three components: two proteins, ParA and ParB, in addition to a *cis*-acting centromere-like *parS* site [30]. ParA is a Walker-type ATPase that interacts with ParB and nonspecific DNA, whereas ParB is a site-specific DNA-binding protein, which recognizes and associates with the *parS* site. Once bound by ParB, low-copy-number plasmids are captured by ParA that, through cycles of ATP binding and hydrolysis, forms dynamic patterns on the nucleoid, in this way moving and eventually positioning sister plasmids in diametrical opposite locations of the dividing cell [31]. ParAB systems encoded by chromosomes are involved in the segregation of newly duplicated origins of replication (*oriC*), adopting dynamics analogous to those described for plasmids and translocating the origins towards opposite cell poles [32]. Recent insights into the role of ParAB in chromosome segregation have been provided particularly by studies on *Vibrio cholerae* [33], *Caulobacter crescentus* 34, 35, 36, 37, 38, *Bacillus subtilis* 39, 40, *Pseudomonas aeruginosa* 41, 42, *Streptomyces coelicolor* [43], and *Myxococcus xanthus* [44].

### Organizing the Chromosome for Segregation: SMC Condensin

Structural maintenance of chromosomes (SMC) proteins are conserved across the three domains of life and mediate crucial chromosome biology processes such as condensation, sister chromatid cohesion, segregation, and DNA repair [45]. The genomes of most bacteria and archaea harbour a single *smc* gene [46]. Most bacterial SMC proteins form a complex with two other factors, ScpA and ScpB. This assembly is also referred to as condensin and plays a key role in compacting the chromosome by bridging and interconnecting DNA loops [32]. SMC condensins are recruited to the bacterial chromosome *oriC* region via interaction with ParB bound to the *parS* sites that are clustered around the origin of replication 39, 40. This organized gathering of condensins at the origin imparts a particular structure to the chromosome and mediates its segregation. Very recent studies have shown that condensins act as molecular ‘staples’ that align chromosome arms in close proximity 47, 48.

## Genome Segregation in Archaea: Potential Suspects and Identified Players

In stark contrast with eukaryotes and bacteria, our knowledge on chromosome segregation in archaea is very rudimentary, partly due to the fact that most archaeal genomes have been sequenced only in the last decade, but also attributable to the development of genetic tools to manipulate some archaea only in recent years. I report the findings of the few investigations conducted so far, discussing implications and questions that still need to be addressed.

### The Role of SMC Condensins

SMC condensins are widespread across archaeal phyla. Early studies analysed the possible involvement of the SMC protein of *Methanococcus voltae* in chromosome segregation. Inactivation of the *smc* gene in this euryarchaeon resulted in aberrant genome partition and cell morphology [49]. Approximately 20% of the cells harboured no chromosome, and around 2% displayed a size that was three to four times larger than that of wild-type cells. Quantitation of the DNA of these so-called titan cells showed a content 10–20-fold higher than that present in normal cells. This phenotype indicates that the SMC protein presides over an important cell-cycle checkpoint in *M. voltae* and plays a crucial role in chromosome segregation [49].

Soppa and colleagues characterized the cell-cycle profile of an SMC-like protein, named Sph1, in *H. salinarum* [19]. Using synchronized cultures, *sph1* gene expression was shown to be cell-cycle-regulated with a peak at the stage of cell division septum formation. Given that maximal expression is reached at a late stage of the cell cycle when chromosome segregation is nearly completed, Sph1 might be involved in DNA repair in a final step of chromosome replication [19]. Whether this SMC factor has a role in genome segregation remains to be elucidated.

Despite the paucity of information thus far, SMC proteins are anticipated to play a significant role in chromosome segregation, based on the high level of conservation and by analogy with the mechanisms uncovered in bacteria.

### The SegAB System: A Hybrid DNA-Partition Machine

**Figure 2:**
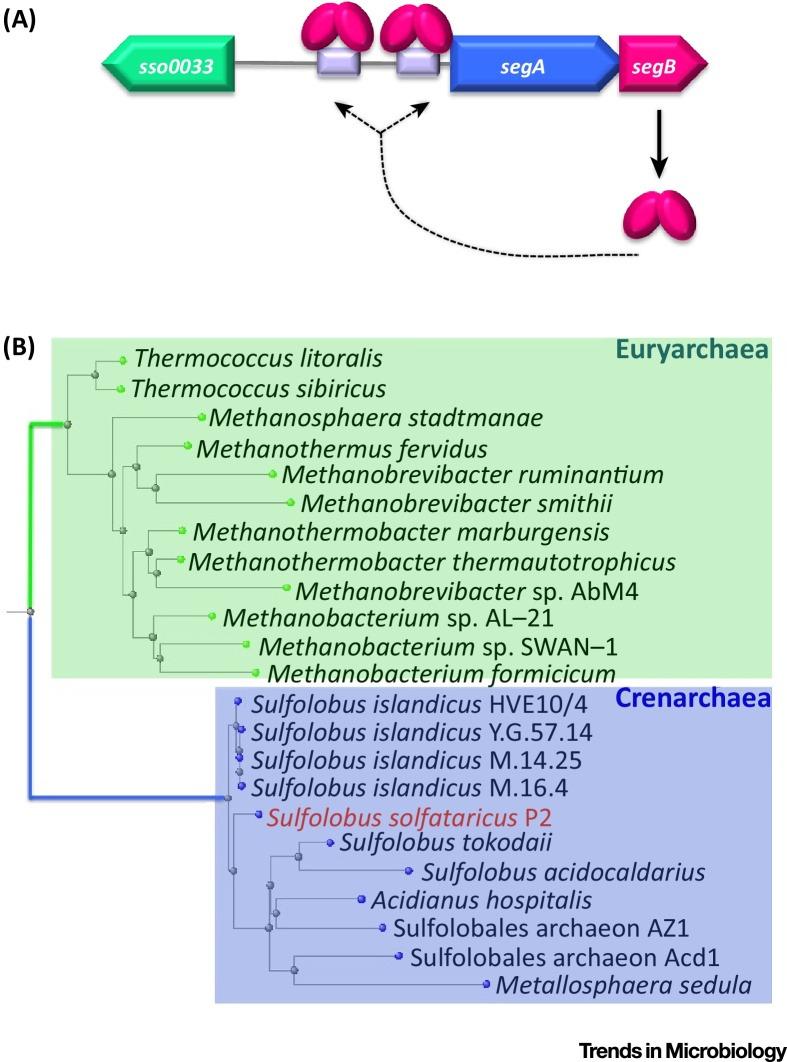
The SegAB System Is Widespread across Archaea. (A) Organization of the *segAB* cassette, including the upstream *sso0033* gene and the two DNA sites (in lilac) to which SegB binds. (B) Phylogenetic tree of a nonexhaustive set of SegB orthologs. Genomic context studies show that each *segB* gene is accompanied by a *segA* gene. Blue box, crenarchaeal SegB cluster; green box, euryarchaeal SegB orthologs. Within the crenarchaeal cluster the *Sulfolobus solfataricus* P2 strain, whose SegAB have been characterized, is shown in red.

A recent study has reported the identification and initial characterization of a dedicated chromosome-segregation system in the thermophilic crenarchaeon *S. solfataricus* [50]. This genome-partitioning apparatus consists of two proteins, SegA and SegB, and a *cis*-acting centromere-like region (Figure 2A). Intriguingly, the complex is a hybrid partition machine: SegA is an ortholog of bacterial, Walker-type chromosome-encoded ParA proteins, whereas SegB is an archaea-specific factor lacking any sequence identity to either eukaryotic or bacterial proteins. However, SegB displays sequence identity to a group of conserved, uncharacterized proteins present in both Crenarchaea (∼80% identity) and some Euryarchaea (30–46% identity) (Figure 2B). Interestingly, the genes encoding SegB proteins are located invariably downstream of *segA* orthologs. BLAST searches against archaeal genomes available so far indicated that the *segAB* cassette is present in an array of archaeal genera belonging to both Crenarchaea and Euryarchaea phyla (Figure 2B). Although the ploidy and genome content has not been determined for all these genera, what is tantalizing is that the majority of these Archaea are monoploid, or at most diploid such as *M. thermautotrophicus*. If a cell harbours only one or two copies of the chromosome, then a rigorous toolkit to segregate DNA at cell division is a stringent *sine qua non*. The 3′ end of *segA* overlaps with the 5′ end of *segB*: this arrangement suggests that the genes may be part of a single transcriptional unit implying that SegA and SegB work together to effect the same biological process. Supporting evidence derives from a transcription profiling study showing that the *Sulfolobus acidocaldarius* homologues of *segA* and *segB* are coexpressed in a cell-cycle-regulated fashion [51].

SegA is an ATPase assembling into higher-order structures *in vitro* upon ATP binding, while SegB is a site-specific DNA-binding protein contacting palindromic sequences located upstream of the *segAB* cassette [50]. The two proteins interact with one another, and SegB synergistically affects SegA self-assembly dynamics, perhaps acting as a nucleator protein. SegB is a dimeric protein that binds specifically to an imperfect palindromic motif located upstream of the *segA* start codon (site 1) and then at position –59 with respect to the same start codon (site 2) (Figure 2A) [50]. These sites might be archaeal centromere analogs. However, at this stage it cannot be ruled out that the sites might also act as regulatory regions that control the expression of the *segAB* cassette. Whether additional sites are scattered across the chromosome is currently unknown.

Microscopy investigations have revealed that increased expression of *segAB* in *S. solfataricus* cells disrupts chromosome segregation, as evidenced by the presence of anucleate cells, highly condensed nucleoids squeezed into one-half of the cell volume and split, guillotined chromosomes [50]. These findings indicate that SegA and SegB play a key role in chromosome segregation. Further support comes from the observations that *segAB* are highly repressed upon UV irradiation [52] and that their expression starts concurrently with the initiation of DNA replication [51], both of which underscore a function in chromosome segregation. The mechanism underpinning how the SegAB complex drives sister chromatids apart remains to be elucidated.

### The AspA–ParBA Machinery: Borrowing Building Blocks from Bacteria and Eukaryotes

**Figure 3:**
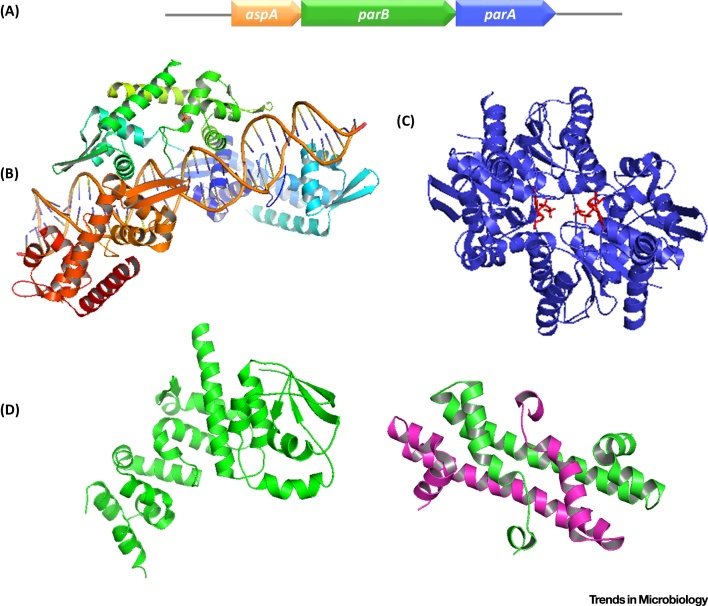
Organization and Structures of the pNOB8 AspA–ParBA System. (A) Schematic diagram of the gene cluster. The 3′ end of *aspA* overlaps with the 5′ end of *parB*, and the 3′ end of *parB* overlaps with the 5′ end of *parA*. (B) AspA–DNA structure (PDB 5FC0) showing three interacting AspA dimers (in orange, green, and blue) associated with the DNA fragment containing the 23 bp putative centromeric site. (C) Adenylyl-imidodiphosphate (AMP-PNP)-bound ParA dimer structure (PDB 4RU8). The ATP analog AMP-PNP is shown in red. (D) (Left) ParB-N structure (PDB 4RSF); (right) ParB-C dimer structure with one monomer shown in green and the other in magenta (PDB 4RS7). The structural images were generated by using PyMOL version 1.8.0.7 (Schrodinger) using the indicated PDB coordinates.

*Sulfolobus* NOB8H2 is a strain isolated by the archaea pioneer Wolfram Zillig and coworkers from acidic hot springs at Noboribetsu in the island of Hokkaido, Japan [53]. This strain harbours a 41 kbp conjugative plasmid, pNOB8, whose sequence has been determined [54]. The plasmid contains ∼50 ORFs including two tandem genes, *orf45* and *orf46*, whose products show homology, respectively, to ParB and ParA families of bacterial partition proteins. The 36 kDa polypeptide encoded by *orf46* is a 315-residue protein with similarity (33–37%) to bacterial ParAs. *orf45* encodes a 470-amino acid protein (55 kDa), whose homology to bacterial ParBs is confined to the N-terminal domain (residues 1–190) (42–58% similarity), whereas the C-terminus shares homology with eukaryotic proteins, including kinesin-like motor proteins. Interestingly, a closer inspection of the region immediately upstream of *parB* revealed a small gene, *orf44*, which encodes a 93-amino acid protein of 10.7 kDa with no sequence homology to any characterized segregation protein [55]. The 3′ end of this gene overlaps with the 5′ end of *parB*, and similarly, the 3′ end of *parB* overlaps with 5′ end of *parA* (Figure 3A). This arrangement suggests that *orf44*, *parB*, and *parA* may be part of a single transcriptional unit. A tricistronic partition cassette is an interesting feature that is not common in the bacterial domain, whose typical segregation modules are bicistronic 30, 31. Furthermore, there is evidence suggesting that the *orf44–parBA* cassette of this plasmid encodes a *bona fide* partition system: when pNOB8 is transferred by conjugation into a different *Sulfolobus* strain, the plasmid undergoes a genetic rearrangement due to a single recombination event, which produces the deletion variant pNOB8-33 53, 54. This plasmid presents a deletion of a ∼8 kbp region resulting in the loss of the *orf44–parBA* cassette and is not stably maintained [53].

A very recent study has provided structural and mechanistic insights into this novel DNA segregation machinery [55]. Orf44, renamed AspA (for archaeal segregation protein A), is a dimeric, site-specific DNA-binding protein that recognizes a 23 bp palindromic motif located upstream of its gene. DNase I footprints have shown that AspA binds to the 23 bp putative centromere and, at higher concentrations, spreads on the DNA in the 5′ direction, protecting over 200 bp from the initial nucleation site [55]. In contrast, ParB binds DNA nonspecifically only at high concentrations, which represents a departure from the bacterial paradigm. The structure of AspA discloses an elongated dimer containing a winged helix-turn-helix DNA binding fold. Remarkably, the AspA–DNA structures exhibit multiple AspA dimers (Figure 3B) that, when extended by packing, lead to the assembly of a continuous left-handed helix.

Interaction investigations established that pNOB8 ParB binds to AspA and ParA. However, AspA does not associate with ParA, suggesting that ParB might act as an adaptor protein within the complex. With 470 residues, pNOB8 ParB is larger than canonical ParB proteins found in bacteria, and consists of two distinct domains, ParB-N (residues 1–320) and ParB-C (residues 370–470) connected by a flexible linker. AspA interacts with ParB-N only, whereas ParA does not bind to either the N- or C-terminus of ParB, suggesting that the extended linker region contains the ParA contacting interface [55]. ParB-C was found to mediate nonspecific DNA binding. The determination of the three-dimensional structure revealed that ParB-N shares weak similarity with the N-terminus of the chromosome segregation ParB, Spo0J, of *Thermus thermophilus* (Figure 3D, left) 55, 56.

Further small-angle X-ray scattering (SAXS) studies on the ParB-N-AspA complex showed that a ParB-N dimer encases the sides and top of the AspA dimer. Interestingly, in the SAXS model, ParB-N dimers can be docked onto each AspA dimer in the AspA-DNA helix, fitting in a lock-and-key fashion into the helix grooves and generating a multiprotein superhelical structure [55]. In this assembly the ParB-C protrudes into the solvent and is connected to the ParB-N domain through the long flexible linker. As ParB-C binds nonspecific DNA, this domain is free to associate with random DNA sequences on either the plasmid or chromosome, or both. Interestingly, this activity indicates that ParB is not simply an adaptor ‘cushion’ sitting between AspA and ParA, but is involved in additional aspects of the segregation process. Surprisingly, the structure of ParB C-terminus exhibits a fold similar to that of the CenpA histone variant which replaces histone H3 on centromere sequences and is involved in assembly of the kinetochore segregation machinery in eukaryotic cells (Figure 3D, right) [57]. This unforeseen observation draws a parallel between DNA segregation in archaea and eukaryotes. A further significance of the finding lies in that, to date, histone homologs have been identified in Euryarchaea; however, they are an exception in Crenarchaea.

The structure of pNOB8 ParA shows strong resemblance to bacterial Walker-type segregation proteins, such as the chromosome ParA homolog, Soj, from *Thermus thermophilus* [58] and multidrug resistant plasmid TP228 ParA homolog, ParF (Figure 3C) [59]. Similar to bacterial ParA proteins, pNOB8 ParA shows nonspecific DNA-binding activity [55]. This finding suggests that ParA might bind the nucleoid in *Sulfolobus* NOB8H2 and thereby might allow anchoring and transport of the plasmid with the aid of ParB. However, the mechanism underlying pNOB8 segregation remains to be elucidated.

Altogether, the AspA–ParB–ParA complex is a novel three-component segregation machine, encoded on both crenarchaeal plasmids and chromosomes, that merges building blocks from bacteria and eukaryotes and opens exciting fresh perspectives on genome segregation in archaea.

## Concluding Remarks

Archaea are ubiquitous inhabitants of our planet. Their ability to thrive in niches where no other organism can survive makes them remarkable objects of investigation for basic studies on life pushed to extremes, but also interesting microbes from which to harness molecules and resources for novel biotechnology applications. The recent identification of Lokiarchaea, a newly proposed phylum with distinctive eukaryotic signatures [60], has rekindled the passionate debate on the origin of Eukarya. However, despite 521 sequenced genomes and a wealth of molecular studies, the fundamental biological process of genome segregation remains a *terra* still vastly *incognita*.

Initial investigations on the cell cycle, nucleoid morphology, and chromosome copy number have laid the foundations for exploring genome partition and highlighted the spectrum of different lifestyles adopted by archaea, when it comes to arranging, condensing, and dispatching their chromosomes. The advent of next-generation sequencing and metagenomics as well as the development of genetic tools to manipulate archaea have allowed the identification of genes encoding components of possible DNA-segregation engines. As observed in other aspects of archaeal biology, a chimaeric nature seems to be at the heart of recently characterized genome-segregation machineries that merge bacterial and eukaryotic elements. How do these archaeal complexes pull DNA molecules apart? And how do these assemblies coordinate their action in space and time with the other closely interlinked cell-cycle events? Do the complexes rely on additional cellular factors? These are just some of the challenges lying ahead and awaiting mechanistic answers (see Outstanding Questions). As we have just started to uncover the tip of the iceberg, other genome-segregation systems undoubtedly exist in the Archaea cosmos, and exciting discoveries are eagerly anticipated for the different phyla. The apparent split between monoploid Crenarchaea and polyploid Euryarchaea is a remarkably interesting biological puzzle that is likely to figure prominently in future trends of the genome segregation field.

Outstanding Questions Do polyploid archaea use an active system for chromosome segregation? If so, what is this system? If not, do these microbes rely on a stochastic diffusion process?

How do halophilic archaea handle the simultaneous segregation of multiple replicons that include chromosome and megaplasmids?

Which and how many genome-partition systems are encoded by different archaea?

How is genome segregation coordinated with DNA replication and cell division in archaea?

Does the archaeal chromosome adopt a specific orientation in relation to reference points within the cell?

What are the characteristics of centromeres on archaeal chromosome and plasmids? Are the centromers clustered in specific positions or dispersed more randomly?

How does the SegAB complex mediate chromosome segregation? How conserved and widespread is this system across Euryarchaea? Are there variations on the SegAB theme in Crenarchaea and Euryarchaea?

Do spherical archaeal cells have functional ‘poles’? If yes, do the poles play a role in chromosome segregation?

What is the mechanism underlying plasmid segregation mediated by the AspA–ParBA assembly? Is pNOB8 chimaeric ParB protein the prototype of a novel family of crenarchaeal histones?
